# How searching under time pressure impacts clinical decision making

**DOI:** 10.5195/jmla.2020.915

**Published:** 2020-10-01

**Authors:** Anton van der Vegt, Guido Zuccon, Bevan Koopman, Anthony Deacon

**Affiliations:** 1 a.vandervegt@uq.net.au, School of Information Technology and Electrical Engineering, University of Queensland, Brisbane, Australia; 2 g.zuccon@uq.edu.au, School of Information Technology and Electrical Engineering, University of Queensland, Brisbane, Australia; 3 bevan.koopman@csiro.au, CSIRO, Canberra, Australia; 4 a.deacon@uq.edu.au, University of Queensland, Brisbane, Australia

## Abstract

**Objective:**

Clinicians encounter many questions during patient encounters that they cannot answer. While search systems (e.g., PubMed) can help clinicians find answers, clinicians are typically busy and report that they often do not have sufficient time to use such systems. The objective of this study was to assess the impact of time pressure on clinical decisions made with the use of a medical literature search system.

**Design:**

In stage 1, 109 final-year medical students and practicing clinicians were presented with 16 clinical questions that they had to answer using their own knowledge. In stage 2, the participants were provided with a search system, similar to PubMed, to help them to answer the same 16 questions, and time pressure was simulated by limiting the participant's search time to 3, 6, or 9 minutes per question.

**Results:**

Under low time pressure, the correct answer rate significantly improved by 32% when the participants used the search system, whereas under high time pressure, this improvement was only 6%. Also, under high time pressure, participants reported significantly lower confidence in the answers, higher perception of task difficulty, and higher stress levels.

**Conclusions:**

For clinicians and health care organizations operating in increasingly time-pressured environments, literature search systems become less effective at supporting accurate clinical decisions. For medical search system developers, this study indicates that system designs that provide faster information retrieval and analysis, rather than traditional document search, may provide more effective alternatives.

## INTRODUCTION

Clinicians routinely raise clinical questions related to their patient interactions that they are unable to answer with their own knowledge [1]. Studies conducted with primary care physicians show that on average, between 0.07 and 1.85 questions are generated per patient encounter, a little under 1 question per hour [2]. Of these questions, many are often left unanswered, as demonstrated by 3 studies in the United States where 63.8% (702/1,101), 44.9% (477/1,062), and 70.2% (207/295) of medical questions raised by clinicians were left unanswered [3–5]. Yet, answering clinical questions correctly has a significant impact on patient outcomes and health system efficiency [6–10].

Medical literature search engines, such as PubMed, provide a means for clinicians to review medical literature while on the job to aid them in answering their questions [11]. Between 75%–80% of clinicians in the United Kingdom, United States, and Canada use PubMed [12]. A number of studies have evaluated the benefit of using such systems to aid in clinical decision making. Westbrook et al. found that the introduction of a medical literature search system significantly improved the correct answer rate from 29% (174/600) without the system to 50% (298/600) with the system [13]. Similarly, Hersh et al. found that a MEDLINE-only search system improved the correct answer rate from 32% (104/324) to 46% (150/324) [14].

In these and other similar studies, time pressure was not a factor in the study. Yet, insufficient time has been identified as the chief barrier to using such medical resources [15, 16]. Moreover, clinicians reported that lack of time and belief that the search system would provide a definitive answer were 2 primary barriers to pursuing an answer [4, 5].

Clinicians are frequently under time pressure as indicated by a range of global surveys. The average primary care consultation times range from 48 seconds to 22.5 minutes. Moreover, in 18 countries—representing around half the global population—the average consultation time is less than 5 minutes [17]. Across 10 industrialized countries—including Australia, Canada, the United States, and the United Kingdom—over one-third of all primary care clinicians reported they were dissatisfied with the time they had available per patient [18]. The British Medical Association's tracker survey, which follows medical staff across the United Kingdom, shows that 68% of general practitioners and 44% of consultants now find their workload unmanageable [19].

This evidence prompts the question: how effective are medical literature search engines (e.g., PubMed) at supporting *time-pressured* clinicians in making better clinical decisions? This study aimed to address this question through the following two specific research questions (RQs):

- RQ-1: What is the impact of time pressure on clinical decision accuracy as a result of using a medical literature search system to support answering clinical questions?
- RQ-2: What is the impact of time pressure on clinicians' perception of (1) answer confidence, (2) task difficulty, and (3) time pressure–induced stress as a result of using a medical literature search system to support answering clinical questions?

Together, these research questions allowed exploration of the impact of time pressure from two perspectives: its objective impact on decision quality and its affective impact on the clinician's state of mind. Both have implications for the efficient and effective delivery of patient care.

## METHODS

The detailed study protocol was previously reported by van der Vegt et al. [20]. Several preliminary trials related to question selection and study timing were conducted with clinicians prior to finalizing the protocol to ensure that participants could successfully complete the study. Ethics approval was granted by the University Human Research Ethics Committee, Queensland University of Technology (approval number 1700000215). No external funding was used for this study.

### Study design overview

**Figure 1:**
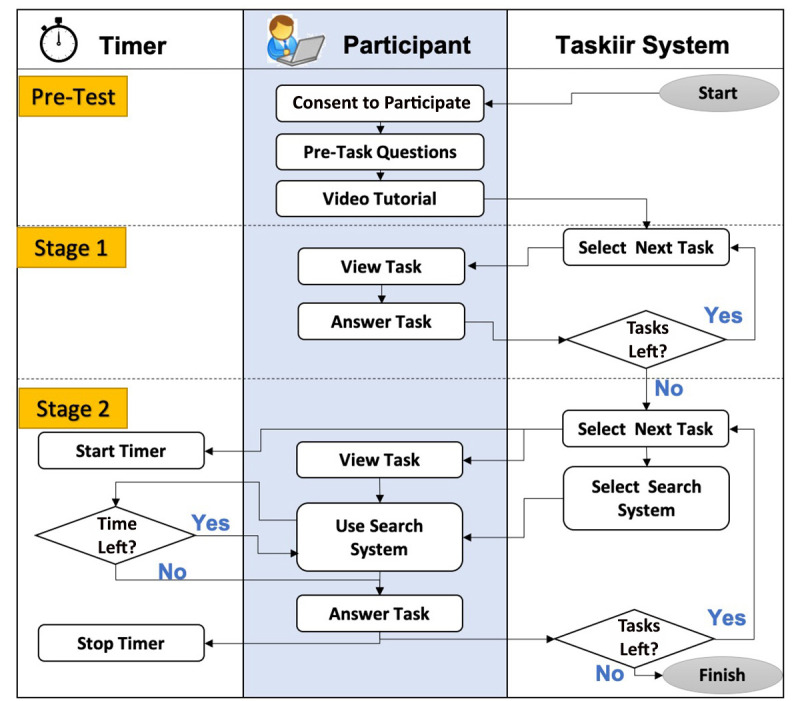
Process flow diagram of the study

Participants were provided with sixteen clinical questions. In stage 1, participants were asked to answer the questions with their own knowledge (i.e., without any supporting evidence) (Figure 1). In stage 2, the same participants were asked to answer the same sixteen questions but were required to make use of a medical literature search system to support their decision making. In this stage, participants' search time for each question was constrained to three, six, or nine minutes to search for suitable evidence.

Stage 1 was not timed, and participants did not use supporting resources. In stage 2, participants used the Taskiir medical search system, with search time constrained to 3, 6, or 9 minutes.

### Participants

A convenience sample of 109 final-year medical students and practicing clinicians—including nurses, general practitioners, and hospital physicians—were asked to participate. The practicing clinician participants had to be registered and residing in Australia. All participants were required to have access to a computer with Internet access. Participants were offered a small honorarium ($50 gift card) to complete the assessment and were recruited via email and online noticeboards directed to clinical departments in hospitals, public health area networks, and medical faculties at Australian universities.

### Procedures

Participants were asked to complete a two-hour, web-based assessment of a medical literature search system called Taskiir. After voluntary consent was received, participants were sent their login details via email. In the email, participants were advised that they could perform the study in multiple sittings, within a two-week period, at times to suit them, and that they must use their laptop or computer (not iPad) to access the study on the web.

After initial login, participants were asked seven questions to capture their demographic data as well as self-reported search and medical experience. A video tutorial followed describing the study in more detail and showed participants how to use Taskiir. The tutorial emphasized that participants had to answer the question without the aid of other people or resources. Once completed, participants were shown specific instructions that again reinforced their obligation to perform the test alone.

In stage 1, 16 clinical questions were presented to participants 1 at a time. Participants were instructed to answer each question within a few minutes, although this time limit was not enforced (mean answer time=34 seconds, standard deviation [SD]=30 seconds, range=7–403 seconds). Fourteen questions required participants to select 1 of 4 answers (“yes,” “no,” “conflicting evidence,” or “don't know”), and 2 questions required a 1–2-word answer. At the end of the last question, the search system moved participants to stage 2 of the study.

In stage 2, participants had to answer the same sixteen clinical questions in the same order as in stage 1. However, participants now had to use Taskiir to help them to answer the question and find evidence to support their answer. Evidence could be collected by participants selecting text and/or images from the source documents they read. The maximum search time allocated to each question was three, six, or nine minutes, based on the assigned timing cohort of each participant. Participants were told of the time allocation at the start of each question, and a minute-by-minute countdown timer was always visible; warnings were given thirty seconds prior to time-out. At time-out, participants were taken to the question completion screen to enter their final answers.

### Time pressure

According to Ordonez and Benson, time constraints exist whenever there is a deadline for a task; however, for the task performer to be time pressured, the time constraint must induce stress so that they feel the need to cope with limited time [21]. In our study, time pressure was induced by specifying and enforcing a time limit for participants to search the literature for an answer to a clinical question. Inducing time pressure with a search time constraint is consistent with previous work in the information search context [22].

In Westbrook et al., the mean time to answer a question was 6.1 minutes (standard deviation=3.1 minutes) [23]. Search time constraints in our study were, therefore, set at the average question answer time (6 minutes) and approximately 1 SD either side of this (3 and 9 minutes). These limits were intended to induce time pressure for 84% of questions with a 3-minute time limit, 50% of questions with a 6-minute time limit, and 16% of questions with a 9-minute time limit. From previous studies, realistic answer time frames for busy clinicians are below 5 minutes, so the 3 proposed search time constraints encompass this pragmatic indicator of search time suitability [24, 25].

Participants were randomly assigned to one of three timing cohorts. The cohort dictated which questions would be performed under which time constraint and was designed to ensure that: (1) the maximum duration of question search time in stage 2 was fixed to ninety-six minutes for all participants; (2) a within-subject design was applied across the time constraint variable such that each participant performed four to six questions per time constraint; and (3) time constraints were applied according to a randomized Latin square approach to avoid confounding effects due to fatigue, time constraint order, and question order.

Search time started when participants landed on the search screen and were shown the question and ended when participants exited the search screen to answer the question. The question timer was stopped while the search system was retrieving documents to eliminate system search time variation or other network or system delays that might have biased the overall available search time. Participants were told that system retrieval time was excluded from the timing to alleviate any additional time stress they might have felt due to a perceived or actual slowness of the search system.

The time constraints were intended to induce time pressure. Time pressure was then inferred through participants' self-reported time pressure level, which they recorded at the end of each clinical question by answering the question, “How would you rate the time you had available to complete this task?” Response options were: (1) not nearly enough time, (2) nearly enough time, (3) just enough time, (4) more than enough time, and (5) much more than enough time. Based on the participant's response, perceived time pressure was then categorized into three levels: high=(1) or (2); medium=(3); or low=(4) or (5).

### Clinical questions

Most of the sixteen clinical questions that participants were asked were derived outside of this study. Six questions were produced and used by Westbrook et al. and consisted of real-life scenarios with a clinical question for each scenario [23]. Westbrook et al. derived these questions using clinical experts and designed them to be clinically relevant and of mixed complexity. Four questions were sourced from Hersh et al. [14]. Three questions were modified from the Text REtrieval Conference 2015, Clinical Decision Support topic set [26]; these questions were provided with diagnoses, which our physician (Deacon) modified into questions in a similar format to the other questions. Finally, our physician devised three other clinical questions for the purposes of this study. To ensure that at least one relevant document existed in the corpus for each question, our physician searched through the corpus, using Taskiir, to identify one or more relevant documents. A sample question is:

A patient staying in hospital had a myocardial infarction two days ago and is now threatening to sign himself out. You suspect this is due to nicotine withdrawal. The patient wishes to stop smoking and seeks your advice on whether he can start nicotine replacement therapy. Is nicotine replacement therapy appropriate for this patient?

Answer=Yes; Source evidence PubMed IDs=[3417926, 3459718]

A full listing of questions and answers can be found in van der Vegt et al. [20].

### Corpus and medical literature search system

**Figure 2:**
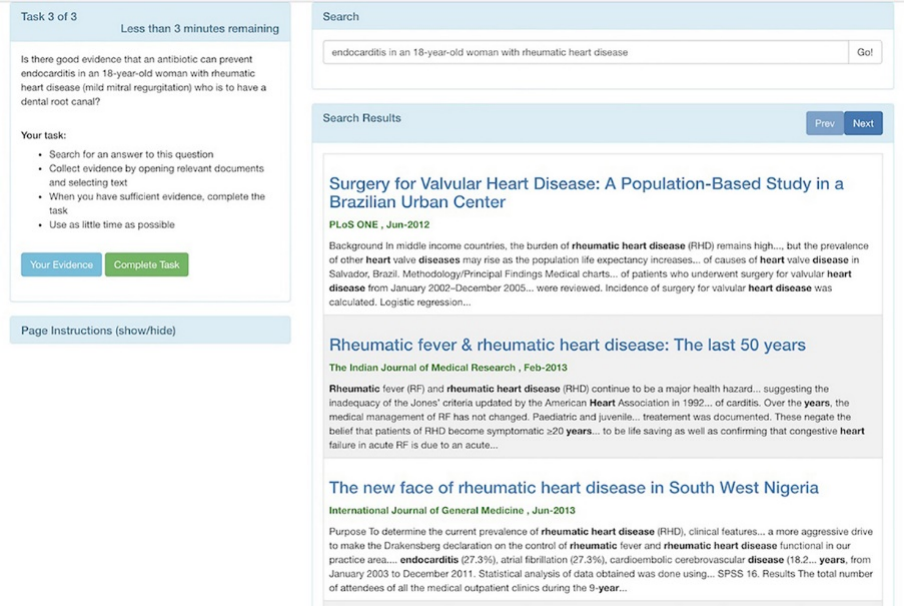
Screenshot of Taskiir, a custom medical literature search engine

A static medical literature corpus and custom search system, called Taskiir, was employed in this study (Figure 2). The corpus of medical documents used was the TREC 2014 and 2015 document collection [26, 27], which consists of a snapshot of the Open Access Subset of PubMed Central taken on January 21, 2014, which comprised 733,138 articles in total. Similar to PubMed and other commercial search engines, Taskiir allowed participants to write their free-text query and perform a best match search of documents in the corpus. A snippet highlighting matching query terms was then provided in the search engine results page appearing below the query. Participants could then select documents of interest to view the full text. While viewing a full-text document, participants could also select (with their mouse) any text or graphics that they wanted to use as evidence for their final answers. Participants could view their evidence or complete the question at any time. Instructions for using the search system were provided on each page.

The box on the left-hand side provided the question, and the search interface was on the right-hand side.

We developed a custom, experimental medical literature search system instead of using an existing search engine, such as PubMed, to ensure full control of the system and because the use of an existing search engine would have exposed participants to a changing document collection over the study time period. The quality of the search system was assessed using standard offline evaluation practices [28] and represents a state-of-the-art clinical literature search [20].

### Statistical analyses

To assess the impact of time pressure on clinical decision accuracy, participants' answers in both stage 1 and 2 were coded as right or wrong by comparing their answers to an expert-judged assessment of each question (provided together with the scenarios in van der Vegt et al. [20]). Stage 2 questions for which no search was performed were discarded, as the value of the search system could not be confirmed in these cases. To assess the statistical significance of changes in the proportion of correct answers between stage 1 and 2, McNemar's chi-square test for symmetry [29] was employed because it was a nonparametric test that was suited for binary variables with samples taken at two points in time. Differences among time constraint categories were analyzed using chi-square tests.

To assess the impact of time pressure on participants' states of mind, a series of questions was asked at the completion of each clinical question as follows, and the resulting data were analyzed using analysis of variance (ANOVA).

1. **Answer confidence** (stage 1 and 2): How confident are you in your answer? (1=No confidence, 2=A little confident, 3=Moderately confident, 4=Very confident, 5=Certain)
2. **Question difficulty** (stage 1 task difficulty): How would you rate the difficulty of this clinical question? (1=Very easy, 2=Easy, 3=Neither easy nor difficult, 4=Difficult, 5=Very difficult)
3. **Search difficulty** (stage 2 task difficulty): How would you rate the difficulty of the search for evidence for this clinical question? (1=Very easy, 2=Easy, 3=Neither easy nor difficult, 4=Difficult, 5=Very difficult)
4. **Stress** (stage 2 only): How much stress did you feel due to time pressure? (1=None, 2=A little, 3=A moderate amount, 4=A lot, 5=More than a lot)

## RESULTS

### Participants

A total of 109 participants (85 final-year medical students, 16 physicians, and 8 nurses) answered 16 clinical questions. Of the 1,744 total number of cases, 85 were discarded because the participant failed to search for the answer, indicating that the search system was not used, and 6 were discarded due to system failure. This left 1,653 cases for analysis.

The gender split of participants was slightly biased toward females (53%), primarily due to a similar split (54% female) among students. Nurse participants were all female, and most (75%) physicians were male. The mean self-reported rating for computer skills was 3.7, with 3 indicating good and 4 indicating very good. Computer skills ratings were lowest for physicians (3.1), slightly higher for nurses (3.3), and highest for students (3.8). The mean self-reported usage of MEDLINE/PubMed was 2.8, with 2 indicating once per month and 3 indicating 2–3 times per month. MEDLINE/PubMed usage was similar for physicians and students (2.9) but lower for nurses (2.1.)

### Research question (RQ)-1: Impact of time pressure on clinical decision accuracy

In stage 1, participants correctly answered 5 out of 16 (31%, SD 1.8, range 0–10) questions on average, which is effectively a random result given the 3 possibly correct answer alternatives. In stage 2, with the aid of a medical literature search system, participants correctly answered 8 out of 16 (50%, SD 2.2, range 0–13) questions on average, representing an overall 20% improvement in the correct answer rate. In stage 1, the correct answer rate did not differ significantly across time pressure cohorts, which was expected given that no time constraint was applied to participants in this stage. However, in stage 2, time pressure was significantly associated with the correct answer rate (χ^2^(2)=66.878, *p*0.001) (Table 1). Specifically, the correct answer rate was 58% higher under low time pressure than under high time pressure (Tukey honestly significant difference [HSD]–adjusted *p*0.001).

**Table 1:** Number of questions answered correctly in stages 1 and 2 and improvement across stages according to time pressure condition

Time pressure	Total number of questions	Correct answers					
		Stage 1		Stage 2		Improvement	
		n	(%)	n	(%)	n	(%)
High	580	178	(30.7%)	238	(41.0%)	60	(10.3%)
Medium	475	168	(35.4%)	261	(54.9%)	93	(19.6%)
Low	598	216	(36.1%)	387	(64.7%)	171	(28.6%)
All	1,653	562	(34.0%)	886	(53.6%)	324	(19.6%)

**Figure 3:**
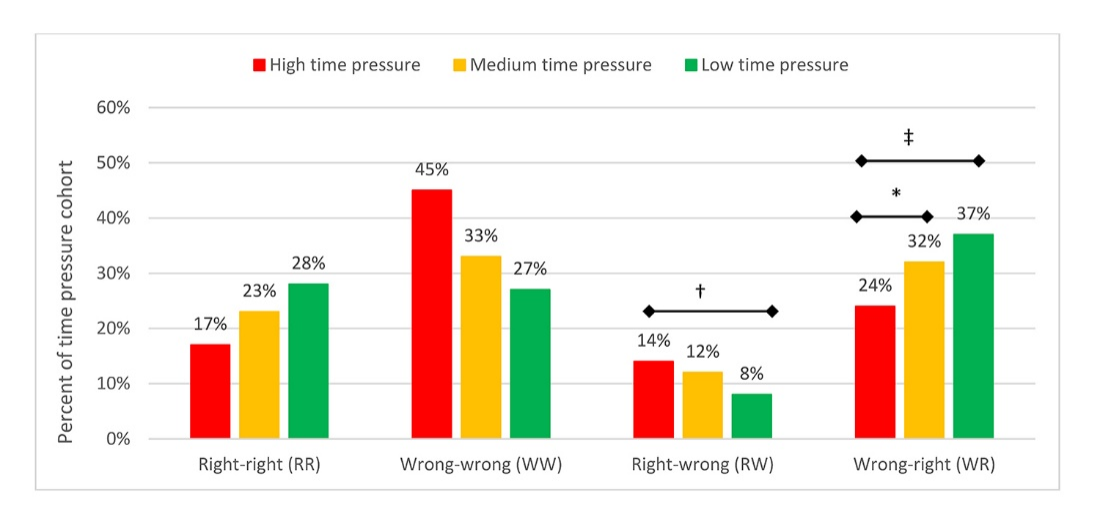
Answer direction between stages 1 and 2 according to time pressure condition

Answer direction indicates the change in answer correctness between stage 1 and 2. For example, the right-to-wrong (RW) answer direction indicates that participants provided the correct answer to a question in stage 1 but an incorrect answer to the same question in stage 2. Overall, time pressure was significantly associated with answer direction (χ^2^(6)=67.877, *p*0.001) (Figure 3). Specifically, the proportion of wrong-to-right (WR) answers was higher under low and medium time pressure than under high time pressure (Tukey HSD-adjusted *p=*0.0052 and *p=*0.0341, respectively). Conversely, the proportion of RW answers was higher under high time pressure than under low time pressure (Tukey HSD-adjusted *p*0.001). In summary, under increased time pressure, participants (1) answered fewer questions correctly, (2) incorrectly changed their answers more often, and (3) correctly changed their answers less often. Medical students, physicians, and nurses showed similar patterns in performance.

Tukey honestly significant difference [HSD]–adjusted *p<0.05, †*p*<0.01, ‡*p*<0.001.

Self-reported computer skills, self-reported frequency of use of MEDLINE/PubMed, and question order were not significantly associated with participants' correct answer rate in stage 1 or 2 or answer direction between stages (chi-square tests, *p*>0.05).

### RQ-2: Impact of time pressure on participants' state of mind

**Figure 4:**
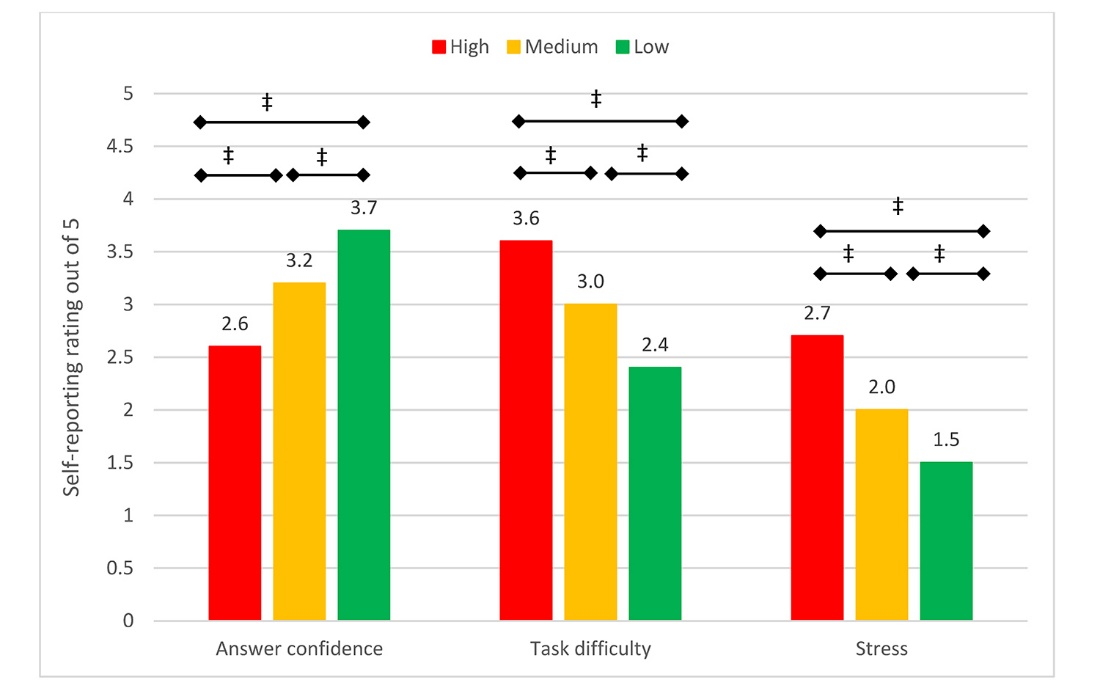
Average self-reported answer confidence, task difficulty, and stress due to time pressure in stage 2

Time pressure had a significant impact on participants' confidence in their answers, perceived task difficulty, and stress (Figure 4). As expected, reported answer confidence (χ^2^(8)=9.6217, *p*=0.2926) and task difficulty (χ^2^(8)=14.078, *p*=0.0798) in stage 1 were independent of time pressure cohort in stage 2. However, in stage 2, lower levels of time pressure were associated with increased answer confidence (χ^2^(8)=376.73, *p*0.001), lower perceived task difficulty (χ^2^(8)=478.25, *p*0.001), and lower stress levels (χ^2^(8)=547.13, *p*0.001). Reducing time pressure from high to low resulted in a 46% increase in answer confidence (Tukey HSD-adjusted *p*0.001), 33% reduction in perceived task difficulty (Tukey HSD-adjusted *p*0.001), and 42% reduction in stress (Tukey HSD-adjusted *p*0.001) across participants.

Tukey HSD–adjusted *p<0.05, †*p*<0.01, ‡*p*<0.001.

## DISCUSSION

We found that the 31% correct answer rate for clinical questions without consulting the literature and the 20% increase in this rate with the use of a medical literature search engine is in line with average rates reported by previous studies [13, 14, 16, 17]. However, these averages concealed the impact of time pressure, which was the key finding of our study.

Under low time pressure, participants doubled their correct answer rate to 65% by making use of a medical literature search system, and they only managed a small improvement to 41% under high time pressure. This suggests that in the time-pressured environments in which many clinicians work, the use of a medical literature search system is hardly helpful. Supporting this finding, under high time pressure, significantly more participants in our study were misdirected by the search system to incorrectly change their answers (i.e., RW answer direction), and significantly fewer participants were able to utilize the search system to correct their incorrect answers (i.e., WR answer direction). To our knowledge, these are the first findings related to the impact of time pressure on clinicians using medical literature search systems to aid their clinical decision making.

Although it was not the aim of this study, trying to understand why participants were more likely to make errors under time pressure is essential if system designers are to improve the effectiveness of literature search engines for time-poor clinicians. One such avenue of potential causality is the impact of time pressure on the clinician's state of mind. Crescenzi et al. found that searchers conducting general exploratory searches under time-constrained conditions reported greater time pressure, felt that the questions were more difficult, and felt less satisfied with their performance [22, 30].

Our study confirms aspects of these findings in a clinical search setting. Further analysis of our data (found in the data repository identified in the “Data Availability Statement”) showed that increased time pressure significantly eroded answer confidence irrespective of whether the participant answered questions correctly using a medical literature search system. Moreover, participants under low time pressure reported greater answer confidence increases between pre- and post-search confidence for correct post-search answers than their high time pressure cohort counterparts. This aligns with a similar study by Westbrook et al. [13] and agrees with psychological literature reporting that increased time pressure decreases decision confidence [31]. This is an important finding for clinical decision making because clinicians' confidence impacts their health care decisions: it reduces the indecision that might arise around selection of treatments and tests and ultimately might lead to a more efficient health care system.

Our findings are consistent with prior work reporting that clinicians prefer the use of manually curated evidence sources, such as UpToDate, over medical literature search systems, such as PubMed [24, 32]. Hoogendam et al. suggest that time to find an answer is a key reason for this preference [24], despite that searching the medical literature can offer benefits over manually curated search systems, including improved currency and breadth of clinical topic coverage.

Medical search systems such as PubMed operate in a reliable, albeit slow, paradigm of information search, in which users have to select and read through documents to find answers. Our results suggest that this paradigm is ineffective or insufficient for time-poor clinicians. Moving from a document retrieval paradigm to a more targeted information retrieval paradigm may present a fruitful direction of research to speed up the search process for clinicians and enable them to take advantage of higher levels of recency and greater breadth of clinical coverage. New search presentation methods, such as faceted search [33] and provision of information cards [34], offer promising research directions in this area. In the meantime, diagnostic systems (e.g., Isabel or VisualDx) may fulfil this role for targeted clinical tasks (e.g., skin cancer identification), and manually curated solutions, such as DynaMed and UpToDate, are likely to be more effective for general clinical search tasks.

This study was devised to control various confounding factors that could jeopardize the results, including question rotations and within-subject timing cohorts; however, despite these intentional safeguards, other variables were outside of the control of this study. First, the study was not conducted under laboratory conditions (i.e., participants did not have to be in a specific location where they could be monitored and the protocol enforced). This meant that despite instructing participants not to use other sources of information to answer their questions beyond our medical literature search system, it was possible that participants did use other sources. Also, participants could talk with other colleagues during the study. Having said this, there was no benefit for participants to veer from the instructions, and, if found out, they would jeopardize their honorarium. Finally, if participants did use other resources, the results suggested that overall time pressure still hampered their decision making or that our results underestimated the true impact of increased time pressure. It was also important to note that evidence suggested that many behaviors did not change significantly when studies were performed remotely rather than in the lab [35].

Second, the same clinical questions were used in both stages 1 and 2. Given more time to ponder the question, participants might have arrived at a correct answer in stage 2 using their own knowledge without the need for search. We felt that this also reflected realistic patient case scenarios, where clinicians might ponder a case and change their thinking. The impact on the study results would be to improve the correct answer rate in stage 2; however, this effect should occur evenly across time constraint cohorts and, therefore, have had minimal impact on the outcome of the research questions.

Finally, it was possible that participants felt greater levels of stress due to use of a new search system rather than due to time pressure. Pre-testing of participant stress levels with the search system was not performed in an effort to limit the overall length of the study. We envisaged that using the search system under the low time constraint condition would represent a reasonable baseline. More importantly, the key findings related to differences in stress levels rather than the absolute levels themselves.

Our findings have significant implications for health care organizations in selecting suitable information systems for their clinicians as well as for medical literature search system designers. The traditional search paradigm of selecting documents from a list of search results and then reading through those documents to find answers may not be effective or sufficient for time-poor clinicians, potentially raising their stress levels, lowering their answer confidence, and reducing their ability to make good clinical decisions. This study provides a basis for further research on medical literature search systems that enable clinicians to find the right information faster. Promising research directions include faceted search, targeted information cards, and summarized results.

## Data Availability

The data underlying our results are available in the CKAN repository at http://ielab-data.uqcloud.net/dataset/timepressure-clinicalstudy.
